# Graham’s Chemistry, with Notes and Additions

**Published:** 1843-11

**Authors:** Robert Bridges


GRAHAM'S CHEMISTRY, WITH NOTES AND ADDITIONS, BY ROBERT BRIDGES, M. D.

The enterprising publishers, Lea and Blanchard, have rendered a great service to American teachers and students of chemistry by the publication of this standard work. As a text-book, it is all that could be desired, and its ample information upon all points within its scope renders it a most valuable work of reference in the laboratory. Its illustrations are numerous and materially enhance its excellence as a manual for students. It is the production of one of the first of living chemists, and exhibits the science of chemistry as it now is, with a judicious fulness of detail, and a luminous clearness which will commend it to the special favor of beginners. Including as it does the applications of the science to the arts, it is adapted to every class of persons who have an interest in chemistry, and we therefore cordially recommend it as one of the very best works extant on the subject. Y.

